# Supplementary material for: Development of Bio-Functionalized, Raman Responsive, and Potentially Excretable Gold Nanoclusters
Source: Nanomaterials (Basel). 2021 Aug 25;11(9):2181. doi: 10.3390/nano11092181 (PMC8471107; doi:10.3390/nano11092181)
Supplement: Supplementary file 1 [file nanomaterials-11-02181-s001.zip › nanomaterials-1352352-supplementary.pdf]

Supporting Materials

Development of Bio-Functionalized, Raman Responsive, and Potentially Excretable Gold Nanoclusters

Ryan D. Mellor, Andreas G. Schätzlein and Ijeoma F. Uchegbu *

School of Pharmacy, University College London (UCL), 29–39 Brunswick Square, London WC1N 1AX, UK; ryan.mellor.16@ucl.ac.uk (R.D.M.); a.schatzlein@ucl.ac.uk (A.G.S.)
* Correspondence: ijeoma.uchegbu@ucl.ac.uk (I.F.U)

Table S1. Materials used in this study.

Material	Supplier	Code
Biphenyl-4-thiol	Sigma Aldrich	752207
Chloroform	AnalaR Norapur	22711.324
Ethanol	Sigma Aldrich	32221
Ethylene glycol bis-mercaptoacetate	Merck	8.14145.0100
Glutathione reduced	ACROS Organics	120000010
Holey carbon TEM grids	EMResolutions	HC300Cu100
Methanesulfonic acid	Alfa Aesar	A13565
Octadecyl amine	Aldrich	74750
Quaternary ammonium palmitoyl glycol chitosan (GCPQ)	Synthesized	N/A
Sodium borohydride	Aldrich	452882
Tetrachloroauric(III) acid trihydrate	Acros Organics	10462971

Table S2. Volumes used in optimization of EGBMA clusters.

Exp	$CHCl_3$ mL	EtOH mL	ODA AuNPs 1 mg mL^{-1} mL	EGBMA 5 μL mL^{-1} mL	Comments
1	3.0	0.5	0.5	1.0	
2	3.1	0.4	0.5	1.0	
3	3.2	0.3	0.5	1.0	
4	3.3	0.2	0.5	1.0	
5	3.4	0.1	0.5	1.0	
6	3.5	0.0	0.5	1.0	
7	3.25	0.25	0.5	1.0	
8	3.45	0.25	0.5	0.8	
9	3.65	0.25	0.5	0.6	
10	3.85	0.25	0.5	0.4	Little precipitation after 24 h
11	4.05	0.25	0.5	0.2	Little precipitation after 24 h
12	4.25	0.25	0.5	0.0	No clustering

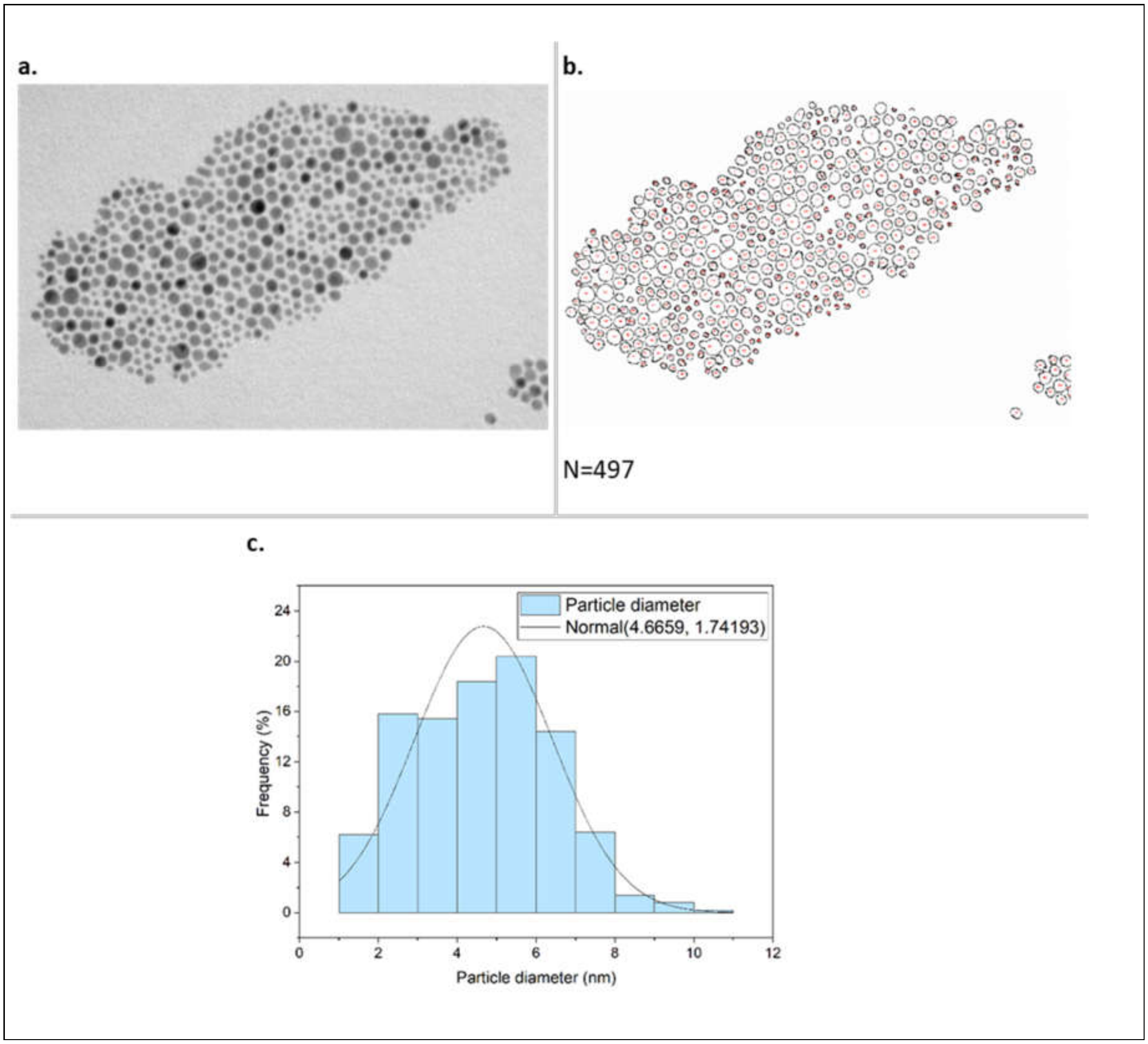

Figure S1. Size analysis of ultrasmalls. **(a)**. TEM image, **(b)**. ImageJ particle identification, and **(c)**. OriginPro distribution analysis.

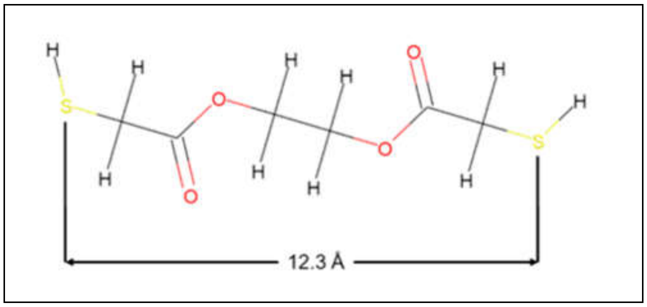

Figure S2. Molecular size of EGBMA.

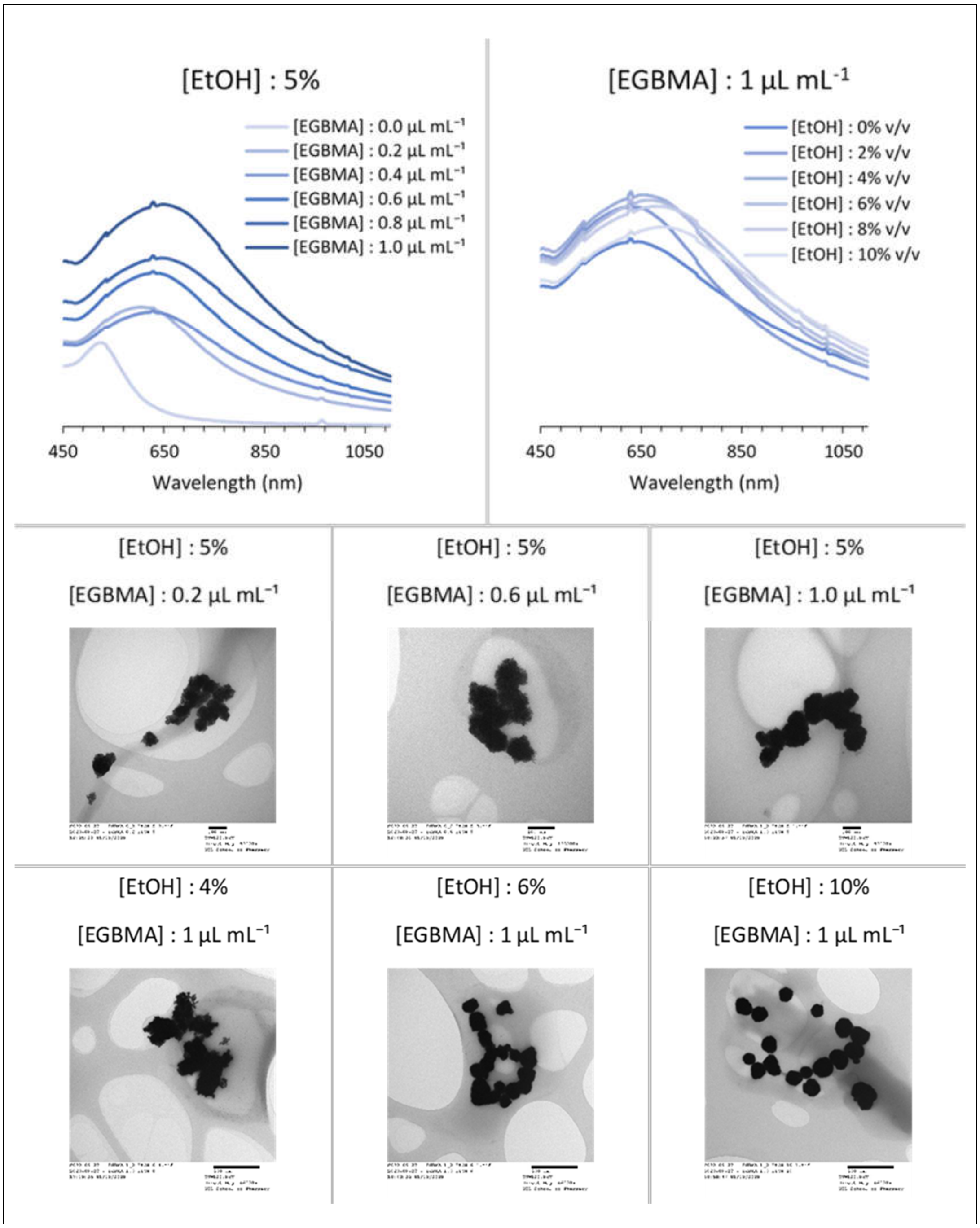

Figure S3. Effect of EGBMA and ethanol concentration on UV-Vis spectrum (top) and TEM images (bottom) of gold nanoclusters.

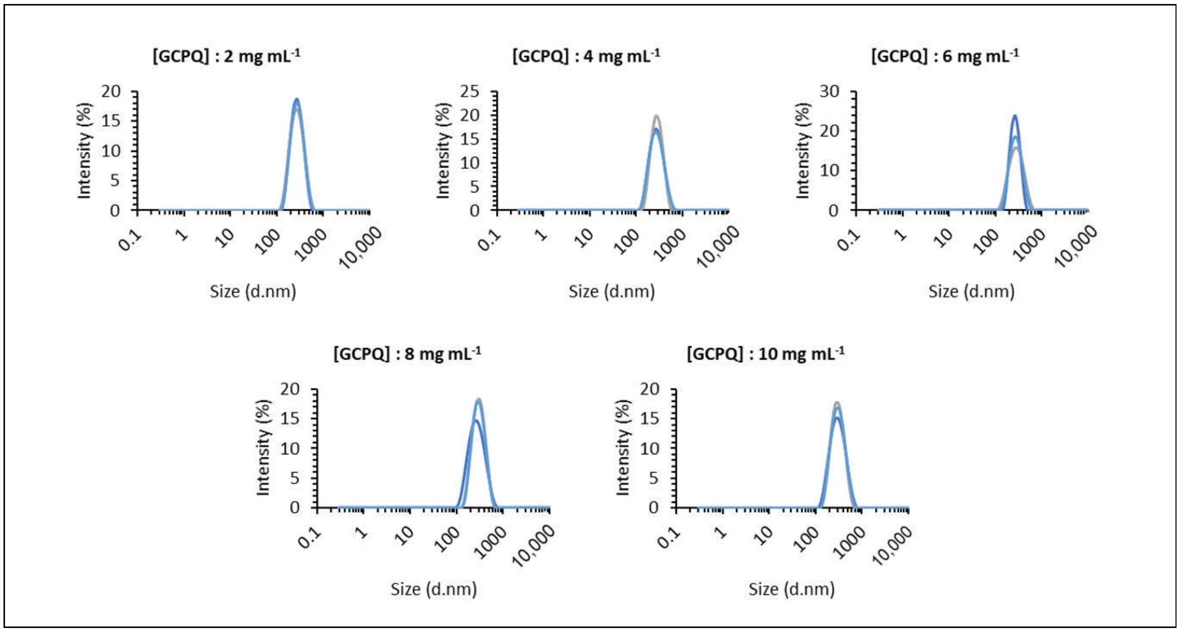

Figure S4. DLS analysis of GCPQ coated EGBMA clusters.

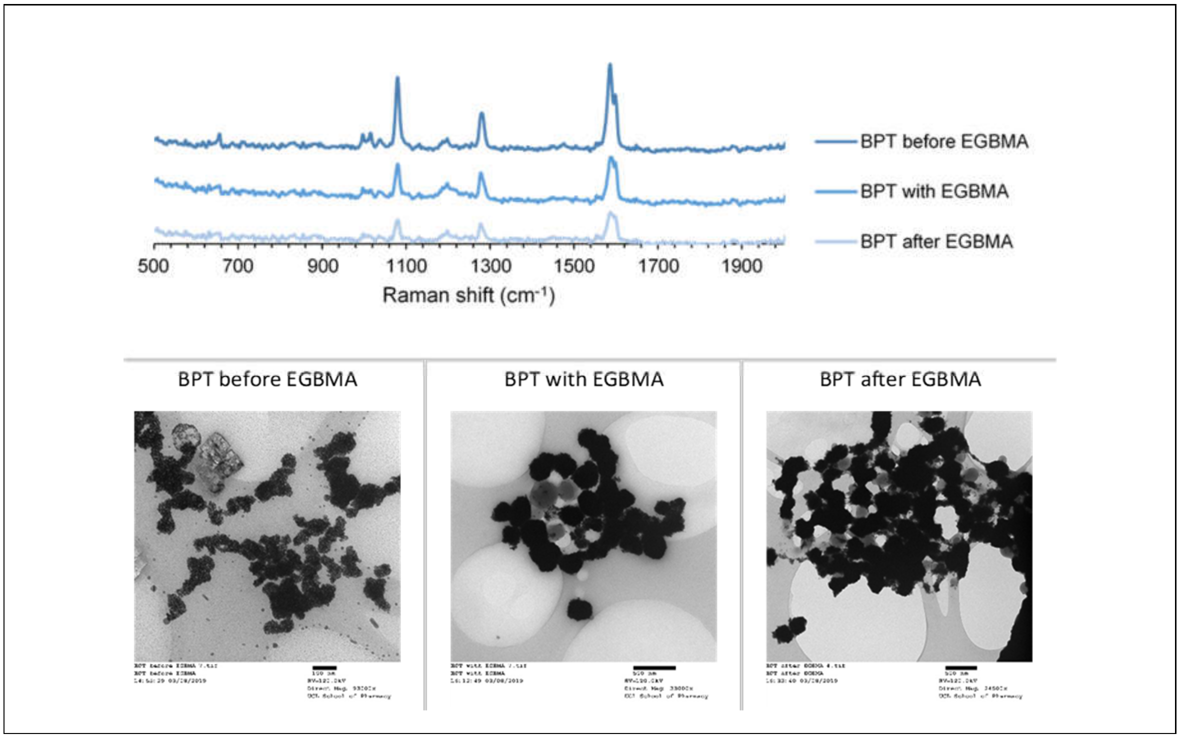

Figure S5. Raman labelling of gold nanoclusters. Raman spectra (top), and TEM images (bottom).

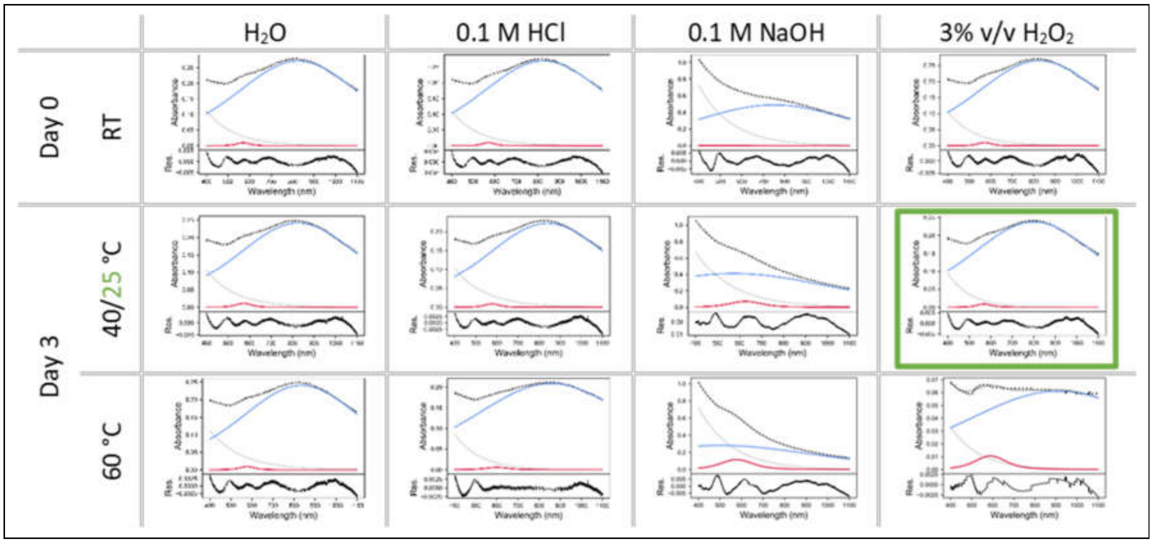

Figure S6. Forced degradation study, deconvolution of UV-Vis spectra. All plots show Rayleigh scattering in grey, ultrasmall component in red, and cluster component in blue.

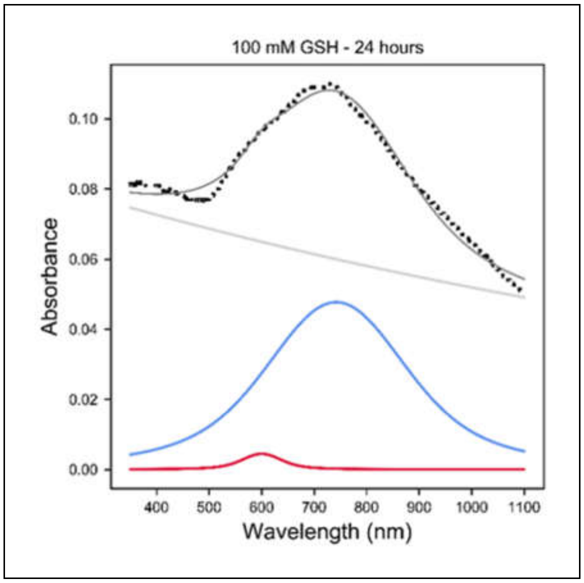

Figure S7. UV-Vis spectra of glutathione (100 mM) degradation of constructs in PBS without pH adjustment.

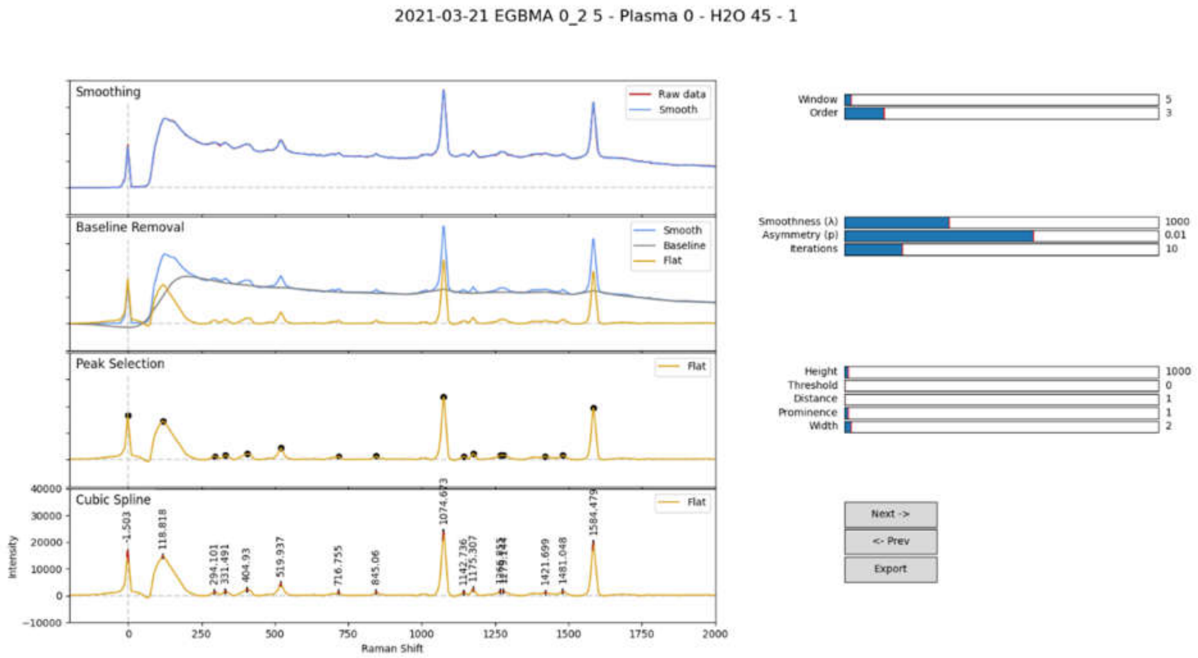

Figure S8. Graphical user interface for processing of Raman spectra.
